# Scene Regularity Interacts With Individual Biases to Modulate Perceptual Stability

**DOI:** 10.3389/fnins.2019.00523

**Published:** 2019-05-28

**Authors:** Qinglin Li, Andrew Isaac Meso, Nikos K. Logothetis, Georgios A. Keliris

**Affiliations:** ^1^Department of Physiology of Cognitive Processes, Max Planck Institute for Biological Cybernetics, Tübingen, Germany; ^2^IMPRS for Cognitive and Systems Neuroscience, University Tuebingen, Tübingen, Germany; ^3^Bernstein Center for Computational Neuroscience, Tübingen, Germany; ^4^Department of Biomedical Sciences, University of Antwerp, Wilrijk, Belgium; ^5^Psychology and Interdisciplinary Neurosciences Research Group, Faculty of Science and Technology, Bournemouth University, Poole, United Kingdom; ^6^Division of Imaging Science and Biomedical Engineering, University of Manchester, Manchester, United Kingdom

**Keywords:** visual perception, bias, bayesian, computational modeling, regularity, psychophysics, human perception, motion perception

## Abstract

Sensory input is inherently ambiguous but our brains achieve remarkable perceptual stability. Prior experience and knowledge of the statistical properties of the world are thought to play a key role in the stabilization process. Individual differences in responses to ambiguous input and biases toward one or the other interpretation could modulate the decision mechanism for perception. However, the role of perceptual bias and its interaction with stimulus spatial properties such as regularity and element density remain to be understood. To this end, we developed novel bi-stable moving visual stimuli in which perception could be parametrically manipulated between two possible mutually exclusive interpretations: transparently or coherently moving. We probed perceptual stability across three composite stimulus element density levels with normal or degraded regularity using a factorial design. We found that increased density led to the amplification of individual biases and consequently to a stabilization of one interpretation over the alternative. This effect was reduced for degraded regularity, demonstrating an interaction between density and regularity. To understand how prior knowledge could be used by the brain in this task, we compared the data with simulations coming from four different hierarchical models of causal inference. These models made different assumptions about the use of prior information by including conditional priors that either facilitated or inhibited motion direction integration. An architecture that included a prior inhibiting motion direction integration consistently outperformed the others. Our results support the hypothesis that direction integration based on sensory likelihoods maybe the default processing mode with conditional priors inhibiting integration employed in order to help motion segmentation and transparency perception.

## Introduction

Our brains are subjected to ambiguous sensory inputs from a variety of sources, yet the world that we perceive appears stable and coherent. To constantly maintain such a percept, dynamic sensory inputs are thought to be combined with our prior knowledge and experience to form what should be consistent neural representations (Knill and Richards, 1996; Rao et al., 2002). Alternative percepts compete dynamically, continuously resulting in changes to the dominant representation driven by interactions taking place at several stages of the cortical hierarchy. Perception can thus vary between multiple outcomes by a myriad of possible mechanisms (Desimone and Duncan, 1995; Beck and Kastner, 2009; Meso et al., 2016b). Biased competition theory suggested that objects simultaneously presented in the visual field compete for neural representation and attention can bias this competition (Desimone and Duncan, 1995; Desimone, 1998; Beck and Kastner, 2009). When stimuli are inherently more ambiguous, such internal processes become more critical in perceptual selection and could govern the outcome of the competition. However, the role of observer bias and how that might interact with key visual stimulus properties which may often control signal strength, remains unexplored. Questions arise following evidence recently found that the human visual system possesses internal templates for regular patterns, indicating that regularity is a coded feature in human vision (Morgan et al., 2012; Ouhnana et al., 2013).

**Figure 1:**
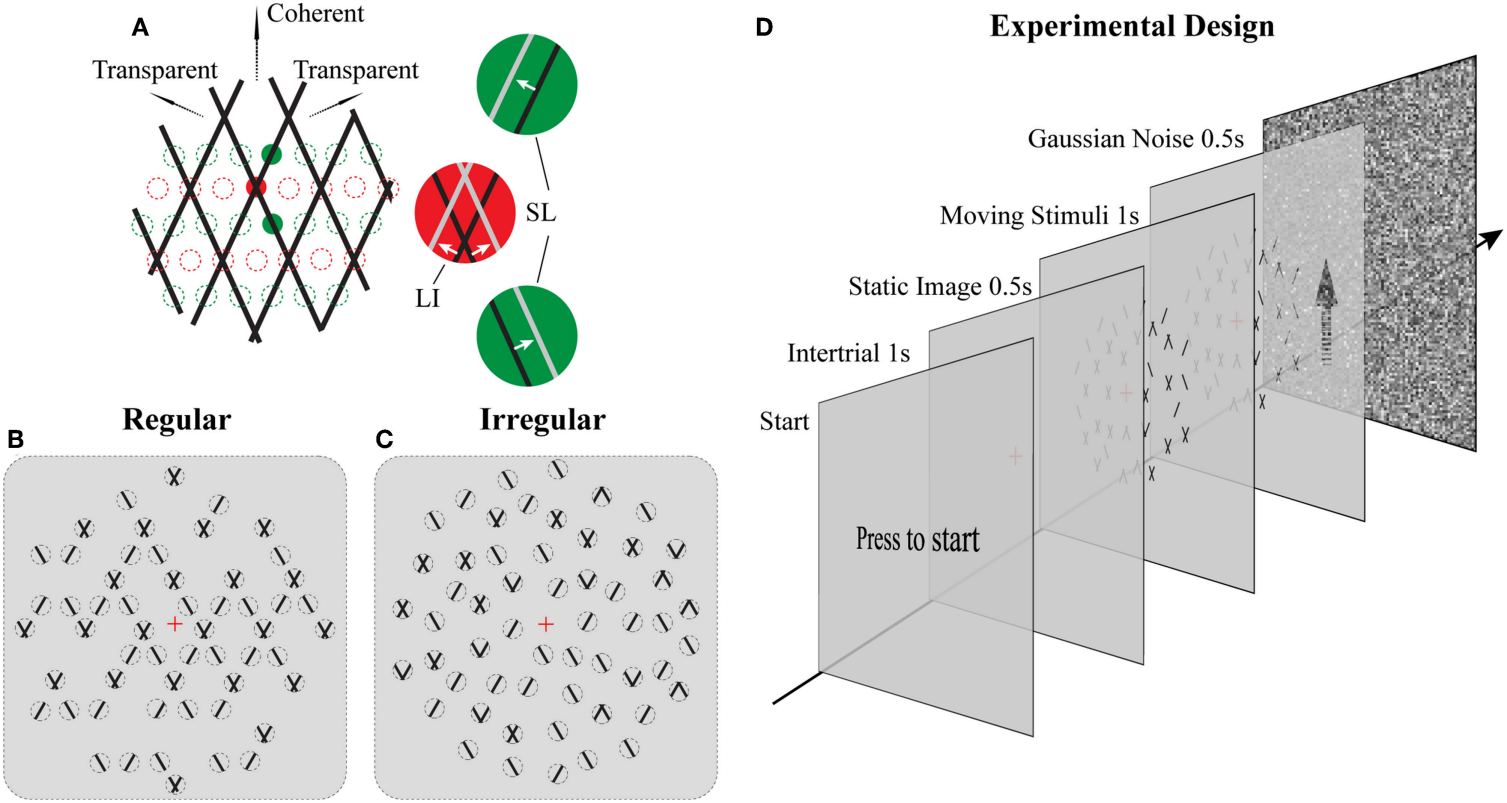
Stimuli and Experimental design. **(A)** Illustration of moving stimuli. The line-plaid (composed of two overlaid drifting line gratings) can be analyzed as containing two different local inputs and namely LI (red) and SL (green). Locations of lines at time t and t + Δt were plotted in black and gray, respectively, in each aperture. Red and green dotted lines indicate exemplary positions of LI and SL apertures respectively. **(B,C)** Cartoon versions of regular and irregular stimuli. In the regular condition **(B)** as the pattern moves up only SI or nothing can appear in SI apertures and likewise for the LI. In the irregular condition **(C)** the aperture locations including their contents were jittered. Dotted lines indicate the locations of the apertures but were not visible **(D)** Experimental design. Subjects had to press a key on the response box to start a trial. After that, a red fixation cross was shown on the center of the monitor for 1 s. The luminance is the same as the mean luminance of the following trial to exclude the influences of luminance changing. A static image of the following trial was presented for 0.5 s to avoid transitional eye movements. After that, the stimulus was shown for 1 s, and subjects had to report their perception by a button press. The trial ended with Gaussian noise presented for 0.5 s to mask potential effects of previous stimuli in subsequent trials.

Here, we developed novel bi-stable visual stimuli (Figure 1) that exploited the significant role of plaid local elements such as intersections (Stoner et al., 1990), to parametrically manipulate perception between two possible interpretations, coherent and transparently moving. We then probed perceptual stability during the resulting ambiguous motion perception across three stimulus density levels with normal or degraded regularity using a factorial design. Further, a set of Bayesian observer models based on the causal inference frame work (Shams and Beierholm, 2010) were developed to perform a perceptual task analogous to the experiments carried out in order to support the investigation of the underlying mechanism. Causal inference has been demonstrated to model perceptual judgements of multisensory integration (Körding et al., 2007; Sato et al., 2007) and fine motion direction judgments done using discrimination (Stocker and Simoncelli, 2007). The approach tackles the problem of having to decide whether two sensory signals come from the same source (in which case they should be integrated) or come from different sources (in which case they should be segregated). These models typically have just four parameters which correspond to the observer's *individual bias* toward one or the other of the of the alternatives; two parameters capturing the *sensory noise* associated with the representation of each competing alternative and finally a *prior width* parameter which defines the extent of the influence the prior has across the measurement space when it is applied. We implement the models in the current experimental context to explore whether performance changes across the density and regularity conditions measured during the tasks are better explained by shifts in one or both sensory likelihood parameters or in prior parameters.

## Materials and Methods

### Participants and Apparatus

Five subjects (college students, four females) participated in all the experiments, four of whom were naïve to the aims of the study. All had normal or corrected-to-normal vision. The study was approved by the ethical committee of the University of Tuebingen. Before data collection, a written participant informed consent was obtained from each subject.

The experiments were performed in a dimly lit room. The stimuli were programmed using Matlab Psychophysics toolbox (Brainard, 1997) and presented on a 17-inch CRT monitor (iiyama, 21sd017) with a resolution of 1,280 × 1,024 and a refresh rate of 100 Hz. The monitor was gamma corrected with a mean luminance of 15.6 cd/m^2^. The distance from the eyes of the subject to the monitor was 43 cm. Responses from subjects were acquired by using a bespoke 2-button response box (see Procedures). Eye movements were monitored continuously using an infrared video eye tracker (iView X^TM^ Hi-speed, SMI).

### Stimuli

The novel plaid stimuli in this study were designed to mimic and manipulate the local elements—lines and intersections—that are carrying the motion signals within the square line plaid stimuli that have been used extensively in psychophysics (Stoner et al., 1990). To achieve this, we decomposed the original plaids into two different types of stimulus patches (see Figures 1A–C; Supplementary Movies 1, 2): separated lines (SL) and line intersections (LI). Although in what follows we refer to these patches as apertures, it should be noted that their dynamic content remained always the same (SL or LI) independent of the position they were plotted. Thus, this allowed us to manipulate the locations of these motion signals to be either consistent with an underlying plaid or jittered in space. The mimicked plaid from which these apertures were created, consisted of two identical superimposed asymmetric line gratings (Hupé and Rubin, 2003; Takahashi, 2004; Moreno-Bote et al., 2010) with a directional difference of 120° (±60 with respect to vertical). Stimulus directions were fixed with respect to the vertical rather than being randomized during the task to avoid previously reported idiosyncratic anisotropies in participant representations of direction (Rauber and Treue, 1999) and to simplify simulated categorical perceptual decisions during the modeling. The spatial frequency of each narrow line grating was 1 cycle per degree, with a duty cycle of 1 pixel or 0.03° and a speed of 2° per second. In order to minimize the luminance effect of the intersection for plaid stimuli (Stoner et al., 1990; Thiele and Stoner, 2003), the luminance of the small intersections remained the same as that of the line. The color of the lines was black (0.9 cd/m^2^) and the background was gray (15.6 cd/m^2^). In Experiment 1 (Regular; Figure 1B) their positions were selected based on a regular grid of locations where either intersections or single lines would be expected in the classic plaid (see positions of red and green dotted circles in Figure 1A). In Experiment 2 (Irregular; Figure 1C), the possible positions of apertures were dynamically jittered vertically from the grid locations (±0.025° of visual-angle) and SL and LI could be located in any of the locations on the underlying grid abolishing the regularity of Experiment 1. The diameter of each aperture was 0.2° of viewing-angle and 720 potential locations were used with no overlap over a stimulus area with a 23°diameter. A rhombus-shaped mask was applied upon each aperture so that no terminators leading to the perception of circular apertures would be seen (Pack et al., 2003). The vertical and horizontal distance between the centers of adjacent apertures was 0.5° and 0.28° of view-angle, respectively. A red fixation cross (0.2° of visual-angle) was shown at the center of the stimuli. No apertures were located within a circular area (2° of visual-angle diameter) where the fixation was centered. The stimuli shared some similarities with previously used multi-aperture stimuli but also had some critical differences (Amano et al., 2009, 2012): (a) within the apertures we used moving lines instead of drifting Gabors, (b) in the regular condition aperture locations for lines and intersections were selected according to the underlying plaid pattern (Experiment 1), (c) the number of apertures was systematically manipulated, and (d) the proportion of different aperture types was used to parametrically change perception.

The total number of apertures was chosen based on three density conditions: low, medium, and high; with 180, 340, and 680, apertures, respectively. New random positions were selected according to these numbers for each trial. In addition, we parametrically manipulated the ratio between SL and LI along 11 homogeneously spaced proportions within the range of 0% to 100%.

### Procedures

For both Experiments 1 and 2, subjects were instructed to press a key on the response box to start a trial (see Figure 1D). After that, a red fixation cross was shown on the center of the monitor for 1 s. Before trial onset, background luminance was slightly adjusted to the mean luminance depending on the density condition to have a homogeneous mean luminance across conditions and trials. First, a static image was presented for 0.5 s to control for transitional eye movements. Then, the stimulus started moving for 1 s, and subjects had to report their perception (either coherent or transparent) during this period by pressing one of two keys. They were instructed to do so as fast as possible and according to their first impression. In order to avoid potential adaptation effects, each trial was followed with a 0.5 s full field Gaussian noise pattern with mean luminance equal to the average of all trials. A method of constant stimuli was used and each psychometric point came from 30 measurements for each of the 11 points along the parametric manipulation of the ratio of the different types of apertures for each subject. All conditions were presented in a pseudo-randomized fashion.

At the beginning of each block, a standard nine-point eye tracking calibration was performed. Subjects took a break after each block. For training, subjects performed 4 blocks of 15 trials before each experiment. They were instructed to fixate the center of the screen and use a chin-rest to avoid head movements.

### Theory and Models

Modeling transparent motion perception presents a challenge of separating unlabeled signals which can come from one source or from multiple sources, posing a computational problem similar to that previously studied with vowel sounds (Sato et al., 2007; Feldman et al., 2009). Here, we used the causal inference framework which originates in multisensory perception and considered the problem to be solved as an explicit two-step hierarchical process with an initial unity vs. separation choice and subsequent direction perception made subject to the influence of the initial decision as a conditional estimate (Stocker and Simoncelli, 2007; Zamboni et al., 2016). This class of models typically has four parameters (Körding et al., 2007; Stocker and Simoncelli, 2007): a participant bias parameter—which we did not use in the current work for reasons explained later, two sensory likelihood parameters corresponding to each alternative sensory representation and a prior width parameter which determines the extent to which the likelihoods can be shifted along the measurement space.

**Figure 2:**
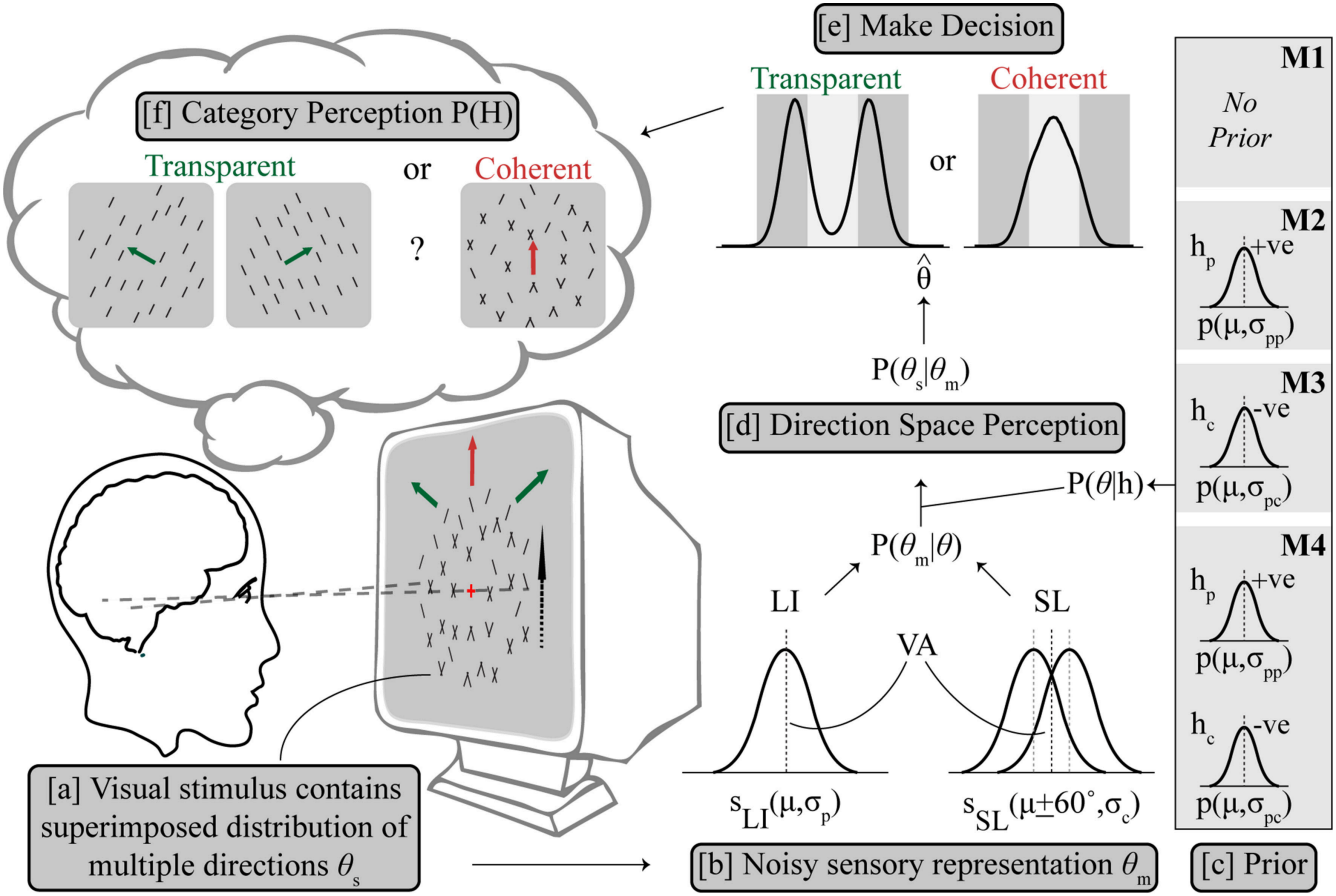
Outline of Bayesian observer model. **(a)** The visual stimulus contains multiple directions of components θ_s_, from which a sensory estimation was made as θ_m_ with uncertainty. **(b)** LI is represented as a Gaussian probability density function (S_LI_) centered on the vector averaged (VA) direction (μ) in the direction space with variance σ_p_, while SL is similarly modeled as two Gaussian probability density functions (S_SL_) centered on μ + 60° and μ – 60° respectively with same variance σ_c_. The likelihood P(θ_*m*_|θ) contains S_LI_ and S_SL_, combining with the respective prior term P(θ|*h*) **(c)** to get the posterior distribution of P(θ_*s*_|θ_*m*_) **(d)**. Prior settings are different for M1–M4, see text for details. The prior terms P(θ|*h*_*p*_) and P(θ|*h*_*c*_) are also both Gaussian terms centered on the VA direction which either enhance (*h*_*p*_) or inhibit (*h*_*c*_) the pattern to support integration or segregation, respectively. **(e)** Decision is made based on a final direction using MAP estimation leading to categorical perception **(f)**.

An optimal Bayesian model would average over the probability of both hypotheses (Körding et al., 2007; Sato et al., 2007), which in this case would be, coherent dominated by components given by *H* = *h*_*c*_ and transparent dominated by the plaid pattern given by *H* = *h*_*p*_, making a decision by reading out from the averaged probability distribution. For a difficult categorical perceptual decision associated with a global percept with mutually exclusive alternatives like ambiguous global motion, we followed previous work (Sato et al., 2007; Stocker and Simoncelli, 2007; Zamboni et al., 2016), and used an implementation in which the optimality of averaging was sacrificed for a quick and self-consistent decision. In other words, a categorical decision is made and this adjusts the shape of the prior probabilities to influence the refined estimate of the second stage. The visual stimulus contains a superimposed distribution of multiple directions of components θ_*s*_, from which a sensory measurement of the perceived direction distribution θ_*m*_, is made by the visual system; an estimate contaminated by Gaussian noise. Given the task at hand in which the alternatives, *h*_*c*_ (components dominate) and *h*_*p*_ (single pattern dominates) cannot mutually exist, we impose an assumption that ambiguity resolution forces the system to commit to one alternative, and its corresponding posterior distribution only, which is either P(θ*|h*_*c*_) or P(θ*|h*_*p*_), illustrated in Figure 2 (Sato et al., 2007).

Three model variants made the following assumptions about the prior: M1 assumed no additional hypothesis about the direction space, i.e., a flat prior with all directions equally likely, then estimation of maximum likelihood P(θ_*m*_) and then categorization of direction; M2 selectively applied a prior on trials where an initial hierarchical step suggested motion integration of the input was needed, consistent with the use of a slow speed prior which has been shown to explain some cases of motion perception (Weiss et al., 2002); The categorical decision in the second step was based on the estimated maximum posterior direction after multiplication with the excitatory prior (*h*_*p*_). M3 similarly computes a categorical decision from the maximum posterior after multiplication with an inhibitory prior (*h*_*c*_) but in contrast on trials which could not be selected by M2, where component separation is suggested by early noisy computations, which supports motion segregation. This novel configuration implements a prior distribution centered diametrically opposite to the average stimulus direction in the circular direction space so that the average direction is inhibited. This is a viable probability distribution configuration in a circular space. Note that for simulations of configuration M2, no segregate priors (i.e., M3) were applied on trials where integrate was chosen and similarly, for the separate simulations under M3 prior no integrate prior (i.e., M2) was applied to any trials. M4 is a control condition which uses either prior (*h*_*c*_ or *h*_*p*_) on each individual trial following the initial estimate, a biologically implausible architecture which we used to allow us to contrast conditions.

The probability of the alternative categorical hypotheses *H*, is given by Equation (1) which includes all the respective likelihoods and priors,

(1) $$\begin{array}{r} P\left(H|\theta_{m}\right)=\;P\left(\theta_{m}|H\right)P\left(H\right)/P\left(\theta_{m}\right) \end{array}$$

Applying model averaging over the posterior distribution (Stocker and Simoncelli, 2007) of each model results in Equation (2):

(2) $$\begin{array}{r} \int P\left(\theta_{s}|\theta_{m}\right)\;d\theta =\;1, \end{array}$$

(3) $$\begin{array}{l} P\left(\theta_{s}|\theta_{m}\right)=\;P\left(\theta_{s}|\theta_{m}\;,\;{H=h}_{c}\right)P\left(H=h_{c}|\theta_{m}\right)\\ \;+\;P\left(\theta_{s}|\theta_{m}\;,\;{H=h}_{p}\right)P\left(H=h_{p}|\theta_{m}\right), \end{array}$$

where the composite posterior in Equation (3) is obtained by adding both alternative posterior probabilities corresponding to each perceptual alternative. We simplify Equation (3) which includes the two separate posterior terms by using model selection to propose an initial fast binary variable computation χ_(1, 2)_, (see simulations) corresponding to hypotheses *H* = *h*_*c*_ and *H* = *h*_*p*_, respectively, to hierarchically separate the early discrimination and the estimation tasks (Luu and Stocker, 2018). In each case, one alternative is selected and the remaining term is set to a probability of zero (Stocker and Simoncelli, 2007). We do not seek an optimal solution to Equations (3) and instead following the lead from previous work sacrifice optimality for consistency (Stocker and Simoncelli, 2007; Luu and Stocker, 2018). During simulations, we assign a decision value of χ = 1, if the MLE is closer to the average (pattern direction) than the component direction, and χ = 2 if the MLE is closer to the transparent component direction (see Figure 4). This heuristic crudely solves the “one vs. two” component problem and reduces the number of free parameters used in this type of experiments from four to three by avoiding the inclusion of a parameter for bias. While individual differences in participant biases have been previously found and modeled (Odegaard and Shams, 2016), in the current work we expected there might be differences within participants across our scene structure conditions and so focused on the interaction between the role of sensory representations and the strength of prior biases. Our heuristic computation of χ similarly constrained all the participants' categorical estimation.

The conditional inference is therefore computed on a given trial according to either,

(4) $$\begin{array}{r} P\left(\theta |\theta_{m},\chi =1\right)=\;P\left(\theta_{m}|\theta \right)P\left(\theta |h_{p}\right)/P\left(\theta_{m}\right), \end{array}$$

in the coherent case where pattern motion is reported or,

(5) $$\begin{array}{r} P\left(\theta |\theta_{m},\chi =2\right)=\;P\left(\theta_{m}|\theta \right)P\left(\theta |h_{c}\right)/P\left(\theta_{m}\right), \end{array}$$

in the case of the transparent choice where the two components are simultaneously perceived. In both Equations (4) and (5), the likelihood term P(θ_*m*_|θ) is identical and contains Gaussian functions of two components and one pattern term whose width captures the sensory noise, and these are shown together as Equation (6).

(6) $$\begin{array}{l} P\left(\theta_{m}|\theta \right)=\frac{A_{S}}{\sqrt{2\pi }}\exp\left(-\frac{{\left(\theta -\theta_{S}\right)}^{2}}{2\sigma_{S}^{2}}\right)\\ \;+\;\frac{A_{S}}{\sqrt{2\pi }}\exp\left(-\frac{{\left(\theta +\theta_{S}\right)}^{2}}{2\sigma_{S}^{2}}\right)+\;\frac{A_{L}}{3\sqrt{2\pi }}\exp\left(-\frac{{\left(\theta \right)}^{2}}{2\sigma_{L}^{2}}\right) \end{array}$$

The average direction of the distribution in Equation (6) is also the pattern direction, θ_*L*_ = 0. The relative scaling of the Gaussian terms corresponding to the alternative percepts is related by *A*_*S*_ = 1-*A*_*L*_. The respective prior terms P(θ|*h*_*p*_) and P(θ|*h*_*c*_) are both Gaussian terms centered on the average direction θ = 0 which either enhance (*h*_*p*_) or inhibit (*h*_*c*_) the pattern to support integration or segregation, respectively. These are given by Equations (7) and (8) and illustrated in Figure 2.

(7) $$\begin{array}{r} P\left(\theta |h_{p}\right)=\;\frac{1}{\sqrt{2\pi }}\exp\left(-\frac{{\left(\theta \right)}^{2}}{2\sigma_{P}^{2}}\right) \end{array}$$

(8) $$\begin{array}{r} P\left(\theta |h_{c}\right)=\;1-\;\left(\frac{1}{\sqrt{2\pi }}\exp\left(-\frac{{\left(\theta \right)}^{2}}{2\sigma_{C}^{2}}\right)\right) \end{array}$$

The prior which acts to enhance the vector average direction of Equation (7) is consistent with a previously proposed slow speed prior which has been demonstrated to explain illusory perception for a range of ambiguous motion stimuli (Weiss et al., 2002). The prior inhibiting the part of the direction space where the average lies is a novel contribution in the current work and is consistent with observations of motion repulsion effects which push direction estimates away from the averages of transparent component directions (Mahani et al., 2005; Meso et al., 2016a). Simulated trials are used to generate psychometric data to study the interaction of sensory motion representations and prior distributions that is most consistent with each participant's performance.

### Simulations

In each trial, assuming a two-step hierarchical process, an MLE estimate based on reduced draws of direction samples of Equation (6) (i.e., 20% of 5,000 used for the full simulation) was used to compute χ based on the distance between the peak of the direction distribution θ_*MAX*_ and the pattern/zero direction. We note that we adopted the convention of making the vertical direction the zero direction so that the component directions flanked this on either side as ±60°. Having fixed directions rather than fully randomizing stimulus directions across space over trials simplifies the process of computing the thresholds of Equation (10). The initial estimation of χ varied with a logistic type non-linear probability as the percentage of LI apertures went from 0 to 100. Slope depended on the likelihood parameters and the PSE (*P* = 0.5) was influenced by the relative widths of the pair of likelihoods. This step captures an implicit categorical decision taken when the stimulus is interpreted at onset using the formulation

(9) $$\begin{array}{r} \theta_{MAX}=argmax\left(P\left(\theta_{m}\right)\right) \end{array}$$

(10) $$\chi =\{\begin{array}{c} 1,\;if\;-\frac{\theta_{L}}{2}<\theta_{MAX}<\frac{\theta_{L}}{2}\;\\ 2,\;if\;\left|\theta_{MAX}\right|>\frac{\theta_{L}}{2}\; \end{array}$$

With χ determined, the posterior of Equation (3) is then simulated using the model selection estimates of Equation (4) or (5) which eliminate the redundant term. Five-thousands draws of direction samples are then used for each trial, binned into a discrete probability distribution with a 0.5° bin resolution. A MAP estimation computes a direction θ_*i*_ for each single trial *i*, from which a second forced choice decision for the simulated trial is made. Transparent or coherent is selected based on the maximum direction (T: θ_*S*_*/2* < |θ_*i*_| or C: θ_*S*_*/2*>|θ_*i*_|) in a similar way to Equation (10). The estimates used to make the categorical decisions assume symmetry across the direction space for simplicity and therefore search for one peak which could be near the pattern direction or within either transparent component, both left and right.

Each simulated trial had a fixed set of stimulus parameters, θ_*S*_ = 60° and θ_*L*_ = 0°. The two sensory likelihood parameters σ_*S*_ and σ_*L*_ along with the relevant prior parameters σ_*P*_ or σ_*C*_ [for M2 or M3] were used to generate psychometric functions for comparison to the empirical psychometric functions for each participant under all six conditions. The best fitting parameters [σ_*S*_, σ_*L*_ *and* σ_*P*_/σ_*C*_] were obtained using an iterative Kullback-Leibler minimization to search the simulated parameter space. Fits to the data were compared across models using Akaike information criterion (Akaike, 1981).

## Results

**Figure 3:**
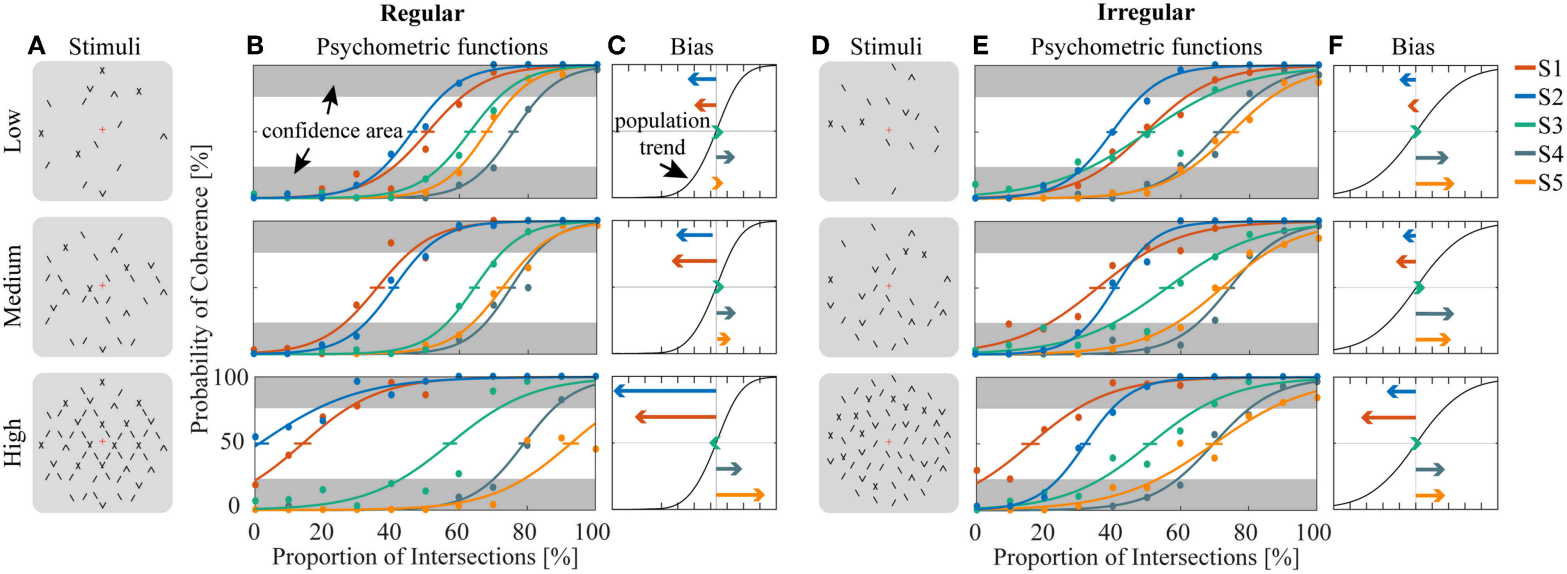
Estimation of bias and stability for regular **(A–C)** and irregular **(D–F)** experiments. **(A,D)** Cartoons of the stimuli across density conditions for the regular **(A)** and irregular **(D)** experiments. **(B,E)** Fitted psychometric functions for each subject across density conditions for the regular **(B)** and irregular **(E)** experiments. The error bar on each psychometric function is the standard error of mean estimated by bootstrapping processing by resampling 400 times. Confidence area (in gray) was defined as where the probability of coherent or transparent perception was higher than 75%. **(C,F)** The direction and amplitudes of bias for each subject corresponding to the conditions of **(B)** and **(E)**, respectively.

**Figure 4:**
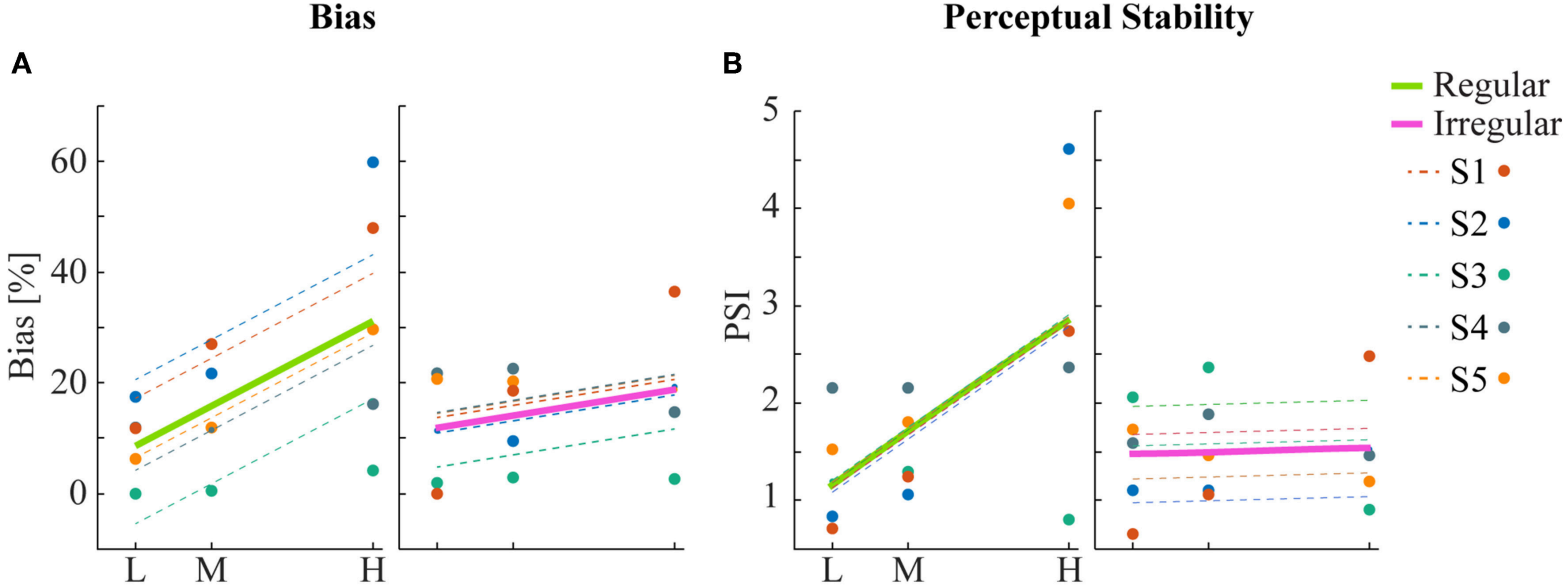
Statistical analysis of bias and perceptual stability. **(A)** Mean bias across subjects for each condition. Linear regression analysis shows a significant correlation between bias and density for the regular but not for the irregular condition. **(B)** Perceptual stability index (PSI, see text) across subjects for each condition. Significant linear correlation between PSI and density was found only for the regular but not for the irregular condition.

Human psychophysics experiments were performed using novel bi-stable line-plaid stimuli (Figures 1B,C). Subjects were instructed to report their perception of either a coherent pattern moving upward, or two transparent surfaces sliding over each other in leftward and rightward oblique directions (see Methods). Inspired by the geometric properties of typically used moving line-plaids (Figure 1A) (Adelson and Movshon, 1982; Pack et al., 2003) and the architecture of the visual system with very small receptive fields (RFs) in early visual areas, we developed this novel stimulus by decomposing the plaid into two types of local stimulus elements we refer to as apertures: separated lines (*SL*) and line intersections (*LI*). In this way, the stimuli could mimic two basic inputs that the visual system could experience locally: 1D- or 2D-motion (green/red apertures, respectively, in Figure 1A) based on the dimensions of the features within the aperture. We performed two experiments with the only difference being the positioning of apertures: in Experiment 1 (regular, R) the structure of the mimicked plaid was maintained (Figure 1B), whereas in Experiment 2 (irregular, I) the element apertures were spatially jittered (Figure 1C). All subjects could consistently fixate within a circular window with radius 0.4 degrees of visual angle (Figure S1). For each subject, we first estimated the relative bias toward one of the two possible percepts (transparent or coherent), by calculating the difference between the 50% coherence threshold taken from its fitted psychometric function and the same threshold calculated from the low-density population trend that was used as a reference (Figure 3). Interestingly, for higher stimulus densities we observed gradual increases in the bias and this effect was more pronounced in Experiment 1 (Regular) in comparison to Experiment 2 (Irregular). Statistical analysis was performed using a linear mixed effects model approach with the bias as independent variable and density and regularity as fixed effects. Subjects were considered as a random effect thus allowing for different intercepts in the model (Figure 4A). Statistical significance was evaluated after parameter estimation using an F-test for the fixed effects with density being significant (*F*_(22)_ = 11.83, *P* = 0.0023) while the interaction between density and regularity remained a trend (*F*_(22)_ = 3.32, *P* = 0.0822). Regularity as a main effect was not significant (*F*_(22)_ = 1.11, *P* = 0.3) indicating that on average the two experiments showed comparable biases.

To obtain a quantitative estimate of the stability of the two percepts for each condition, a perceptual stability index (PSI, Figure 4B) was calculated for each subject as follows: first, we defined as perceptually stable the stimuli that resulted in either coherent or transparent perception with probability over 75% (i.e., see the shaded areas in either side of the psychometric curve with *P*_coherent_ < 25% or *P*_coherent_ > 75% in Figure 3). Then, the PSI was calculated as the fraction of fitted data-points within the side of the confidence area corresponding to the dominant percept, and the rest of the points (Figure 4B). Similar linear mixed effects modeling analysis as for the bias was then performed with the PSI as independent variable. The results showed a significant main effect of density (*F*_(22)_ = 6.38, *P* = 0.0193) as well as significant interaction between density and regularity (*F*_(22)_ = 5.55, *P* = 0.0278). Regularity as a main effect was not significant (*F*_(22)_ = 1.88, *P* = 0.18).

To study the relative contribution of prior experience and sensory representation to the processing of the ambiguous motion direction, we modeled the underlying motion perception task using a Bayesian causal inference framework (Sato et al., 2007; Stocker and Simoncelli, 2007; Shams and Beierholm, 2010). To this end, we used models of increasing complexity (no prior, a transparent prior or a coherent prior, and as a control a model with the use of both priors). In the simplest model architecture (M1, no prior), the maximum likelihood was estimated and categorized depending on whether it was closer to the coherent or transparent direction. For models M2 and M3, a hierarchical sequential computation was assumed and on each simulated trial an initial noisy direction estimate χ, was used to determine whether to apply an excitatory (M2, run as a separate independent simulation from M3) or an inhibitory (M3, run separate from M2) prior, each of which required a single additional Gaussian width parameter centered on the average direction. These would have an effect of shifting posterior probabilities to bias perception either toward coherent (M2) or transparent (M3). Last, in a control condition, a model M4 was simulated by using the best fitting M2/3 parameters and therefore included separate optimal priors for separation and integration. Motion direction was represented as a linear combination of Gaussian probability density functions representing the LI and SL aperture direction and variance (Figure 2; also see Methods). The set of models, M1–M4 were tasked with a forced choice decision on whether each simulated trial corresponded to transparent or coherent, over a number of conditions recreating Experiments 1 and 2.

**Figure 5:**
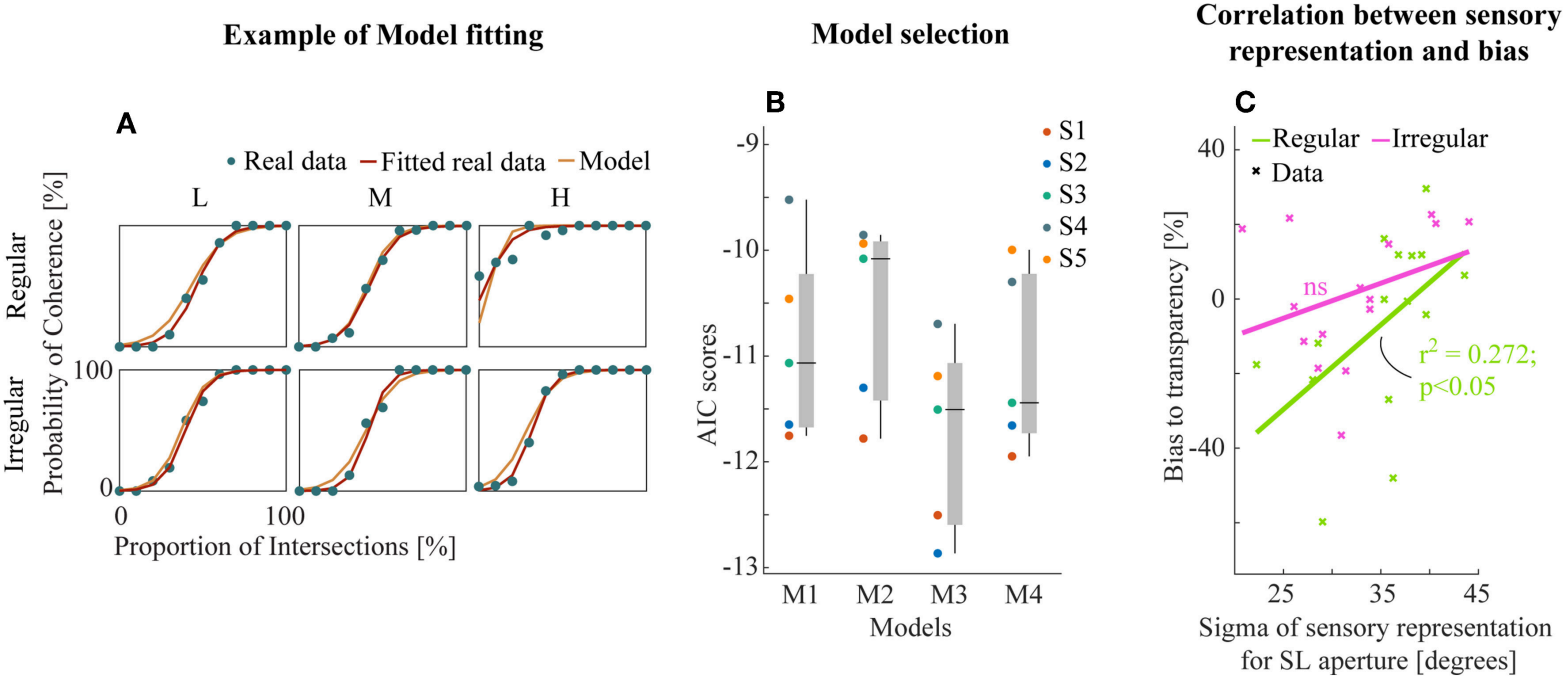
**(A)** Example model fitting results from a representative subject. Empirical and model simulated psychometric functions were plotted for each experiment and conditions. **(B)** The raw AIC scores of all models (see text). Each subjects (left) and box plot (median ± s.e.m., right). **(C)** Linear correlation between the sensory representation of SL aperture and the amount of bias to transparent perception. Data points were collapsed across all density conditions of regular/irregular experiments.

Example model-fitting results for a representative subject are shown in Figure 5A (results for all subjects in Figure S2). We then performed model comparison based on the Akaike criterion measures (AIC, Akaike, 1981) to identify the optimal model architecture. The AIC measurements use likelihoods from the fitting residuals to determine which model provides the best explanation for the data, giving a lower score for better fits but penalizing models with more parameters. M3 (transparent prior) was found to be the most appropriate model for the data set based on AIC scores (Figure 5B). This suggests a general tendency within the visual system toward separating motion components unless there is strong sensory evidence for integration into a single object (here provided by the line intersections (LI) apertures).

Further, we analyzed the relationship between the best model parameters of M3 and perceptual bias from empirical data to investigate the potential insights into sensory mechanisms of subjective biases. We found a significant linear correlation between the bias and the variability of sensory representation (Gaussian likelihoods) for SL apertures (*r*^2^ = 0.272, *p* < 0.05, Figure 5C) only for the regular experiment suggesting that regularity influences the effectiveness of the sensory representation by decreasing variance. There were no similar trends in the fitted parameters for LI sensory likelihoods and the prior (Figure S3).

## Discussion

In this study, we used bi-stable motion perception as a tool to understand processes of perceptual stabilization in the human brain. We used a Bayesian causal inference framework (Sato et al., 2007; Stocker and Simoncelli, 2007; Shams and Beierholm, 2010) to model the internal decision process leading to one of the two alternative interpretations with the aim to understand the relative role of priors and sensory evidence in the selection process. We found, counter-intuitively, that adding more motion information by increasing the number of apertures increased response biases in the task. Individuals' tendencies to either one or the other of the percepts were amplified substantially when we increased the density of stimulus apertures. This led to an increased inter-subject variability, with each subject diverging from the population trend with a magnitude and direction that was related to their original bias (Figure 4A). Interestingly, this effect was largely abolished in the irregular condition when the position of elements was jittered with respect to their original location, indicating that this form of contextual organization created by spatial regularity played a major role in the amplification of the bias. As a measure of the effect of bias amplification, we computed a perceptual stability index and found that it linearly increased for higher element density.

To further understand the brain processes leading to this result, we adapted hierarchical motion perception models that posit sequential stages of brain processing including local motion detection, global combination of these local signals and then an interpretation of the representation to support categorical/qualitative decisions. This broad mechanistic view is widely supported by evidence in the literature for both psychophysics and physiology (Burr and Thompson, 2011; Nishida, 2011). In the context of our work, the representation of the local motion information can be reflected directly in the neural responses in directionally selective areas such as MT/MST, however, one of the classic difficulties of motion transparency perception is how such a local representation can be transformed into the qualitative percept (e.g., see Qian et al., 1994; Treue et al., 2000; Meso and Zanker, 2009). To this end, and in particular with respect to prior information encoded in the brain of each participant, we built a battery of Bayesian models (M1–M4; see Methods) with the task to probabilistically select one of the two percepts on a trial-by-trial basis simulating the experiments. These modeled the sensory representations of the 1D- and 2D-motion input-signals as Gaussian processes each with separate sigma likelihood parameters and, in addition, one of four different prior probability configurations. M3 (which included a segregation prior) provided the best model, suggesting that the visual system selectively applies an inhibition within the direction space to help separate components. Importantly, it should be noted that M3 was the better model even in subjects that were biased toward coherent percepts. We conjecture, that the brain when faced with such tasks applies a conditional implementation of separating priors on some critical trials (Zamboni et al., 2016) and not an integrating one because integration might arise naturally from overlapping signal distributions (Mahani et al., 2005). The proposed hierarchical computation extends recent findings in which participants performed an orientation discrimination followed by an orientation estimation task, with the discrimination found to influence the estimation task (Luu and Stocker, 2018). A similar effect had been found for motion stimuli (Zamboni et al., 2016) with a need for self-consistency proposed as an explanation. We argue that this hierarchical two-step computation might occur during our task, with an implicit early categorical decision needed to resolve the ambiguity resolution known to occur early in motion stimuli (Meso et al., 2016a). In the implemented model, for simplicity, fixed directions were explicitly associated with the categorical decisions. Similar models could be implemented in the future in which, the decision need not be based on the absolute directions but reached based on the distribution of global motion directions after pooling (i.e., a bimodal distribution would signify transparency and a unimodal coherence). In that case, the future tested priors could be adjusted and made independent of direction for example by acting broadly as an attractor or repellant of nearby directions.

Bias stands at the core of signal detection theory (SDT) when applied to both living organisms and machines. In fact, (Green and Swets, 1966), being the first to develop SDT approaches in psychophysics, directly criticized previously used methods for not being able to separate the sensitivity of subjects from their potential biases. In addition to the principle problem of detecting signal within noise, our brains also face the problem of inherently ambiguous sensory inputs. Thus, to make veridical interpretations of the outside world, the brain needs to employ additional mechanisms such as attention and prior experience (Knill and Richards, 1996; Desimone, 1998; Rao et al., 2002; Beck and Kastner, 2009; Meso et al., 2016a). One theory suggested that objects simultaneously presented in the visual field compete and attention can bias the outcome of this competition (Desimone and Duncan, 1995; Desimone, 1998; Beck and Kastner, 2009). Our results are consistent with the general framework of the biased competition hypothesis; however, attention does not seem to be the primary source of the observed biases as there is no reason to expect attention to vary systematically across the different density or regularity conditions. The subjects had to continuously perform the task of reporting their percepts in randomized trials within blocks so attention should have remained largely constant. Moreover, individual bias directions were independent of the stimulus configuration (which was the same for all subjects) precluding bottom-up stimulus driven attention effects. The subject specific results suggested a strong influence of prior experience or assumptions and thus we expected our modeling results might reveal that some subjects would use a “coherence” prior (M2) while others a “transparency” prior (M3). To our surprise, M3 (in comparison to M2; Figure 5B) was a better model for all our subjects, including those with biases toward coherence. This suggests that the sensitivity of the visual system of each participant to the two motion signals (sensory σ) was more important for determining bias direction in comparison to the integration prior. We conjecture that motion direction integration based on sensory likelihoods maybe the default processing mode with conditional priors inhibiting integration employed in order to help motion segmentation and transparency perception.

Furthermore, bias in our experiments was increased with stimulus element density. This was also an unexpected finding, as previous studies have shown that increases in the density of random-dot-kinematograms (RDKs) result in coherence thresholds also decreasing (Barlow and Tripathy, 1997) or being unaffected (Eagle and Rogers, 1997; Talcott et al., 2000; Welchman and Harris, 2000). We note, however, that RDK experiments are closer to the foundations of SDT (i.e., detecting signal within noise). We propose that in our scenario, competition between the two motion representations may be enhanced by density increments resulting in the observed increase of the bias toward a preferred representation which would act like a perceptual attractor, an area within the direction space where probability increases at higher densities. This is consistent with reports in previous literature where contrast-based motion signal increases resulted in stronger 2D motion attractors compared to 1D directions in a tri-stable ambiguous motion stimulus (Meso et al., 2016b). In addition, research with RDKs demonstrated that coherence thresholds in 5–6-year olds were (a) much higher, and (b) decreased with dot density in comparison to adults (Narasimhan and Giaschi, 2012). In our view, this provides evidence for coherent perception or integration as the earliest unelaborated default computation and with perhaps the connectivity of the underlying neural circuitry prone to changes by experience during development. This could explain the different directions of the biases in different subjects.

Interestingly, the bias-amplification and the increases in the perceptual stability index with density were largely abolished in the irregular stimuli with jittered aperture positions. This is consistent with previous work demonstrating the importance of regularity (Morgan et al., 2012; Ouhnana et al., 2013) which appears to play a role in the selection of stable neural representations. Another interpretation is that reduction of regularity eliminates in parallel the correspondence of the single stimulus elements to the underlying patterns or “objects,” interfering with their spatial integration. This is consistent with studies that have demonstrated a precedence of global features in visual perception (Beck and Kastner, 2005; Phillips et al., 2015; Ding et al., 2017). Moreover, the profound influence of position jitter on the bias indicates that the scale of the integration cannot be completely local nor global as in that case the regular/irregular conditions should not elicit an effect. These results directly indicate that the motion integration mechanisms contributing to individual biases are of “meso-scale” i.e., go beyond single-neuron receptive fields (RFs) in V1 to scales more typical for area V5/MT but not the very large RFs found in size-invariant object selective areas like inferotemporal cortex (IT).

Previous research has found strong evidence for active perceptual stabilization mechanisms in the visual system, such as reorganization of sensory representation during intermittent viewing (Leopold et al., 2002); top-down modulation of beta-band synchronization (Kloosterman et al., 2015); feedforward inhibition (Bollimunta and Ditterich, 2012) arousal (Mather and Sutherland, 2011; de Gee et al., 2014); and memory (Wimmer and Shohamy, 2012). Our study suggests that bias serves as an additional factor our brains actively use to stabilize our perception of the world.

## Ethics Statement

This study was carried out in accordance with the recommendations of the ethical guidelines, University of Tuebingen with written informed consent from all subjects. All subjects gave written informed consent in accordance with the Declaration of Helsinki. The protocol was approved by the ethical committee of the University of Tuebingen.

## Author Contributions

QL and GK conceived and designed the psychophysics experiments. QL performed psychophysics experiments and analyzed all data. AM developed the models. QL, AM, and GK run model simulations and wrote the manuscript. NL supported the study and provided experimental equipment. GK supervised the study. All authors interpreted the experimental results and contributed to the final manuscript and gave final approval for publication.

### Conflict of Interest Statement

The authors declare that the research was conducted in the absence of any commercial or financial relationships that could be construed as a potential conflict of interest.

## Supplementary Material

Supplementary Figure S1 Eye movement results. Each subplot shows the averaged eye movement results of each subject from regular/irregular conditions.

Supplementary Figure S2 Psychometric functions. Empirical and simulated psychometric functions were plotted for each experiment and condition.

Supplementary Figure S3 The amount of bias of transparent perception is not correlated with sensory representation of LI aperture (regular condition: r: −0.43, p: 0.10; irregular condition: r: 0.10, p: 0.70), nor with prior (regular condition: r: −0.14, p: 0.60; irregular condition: r: 0.06, p: 0.81)

Supplementary Movie 1 Regular stimuli with representative three density conditions (100, 50 and 0% of LI from in total 500 apertures each, the same as Movie 2).

Supplementary Movie 2 Irregular stimuli. Note that the demo movies were not used for real experiments.
