# Supplementary material for: Scene Regularity Interacts With Individual Biases to Modulate Perceptual Stability
Source: Front Neurosci. 2019 May 28;13:523. doi: 10.3389/fnins.2019.00523 (PMC6546877; doi:10.3389/fnins.2019.00523)
Supplement: Supplementary Figure S1 — Eye movement results. Each subplot shows the averaged eye movement results of each subject from regular/irregular conditions. [file Data_Sheet_1.PDF]

Supplementary Material

Scene regularity interacts with individual biases to modulate perceptual stability

Qinglin Li[1,2,3,6,♠,*] Andrew Isaac Meso[4,♠], Nikos K. Logothetis[1,5] and Georgios A. Keliris[1,3,6,*]

1. Department of Physiology of Cognitive Processes, Max Planck Institute for Biological Cybernetics, Tuebingen, Germany
2. IMPRS for Cognitive and Systems Neuroscience, University Tuebingen, Germany
3. Bernstein Center for Computational Neuroscience, Tuebingen, Germany
4. Psychology and Interdisciplinary Neurosciences Research Group, Faculty of Science and Technology, Bournemouth University, UK
5. Division of Imaging Science and Biomedical Engineering, University of Manchester, Manchester M13 9PT, United Kingdom
6. Department of Biomedical Sciences, University of Antwerp, 2610 Wilrijk, Belgium

♠ Equal contribution

* CORRESPONDENCE:
Prof. Dr. Georgios A. Keliris
georgios.keliris@uantwerpen.be
or
Dr. Qinglin Li
qinglin.li@tuebingen.mpg.de

Supplementary Figures

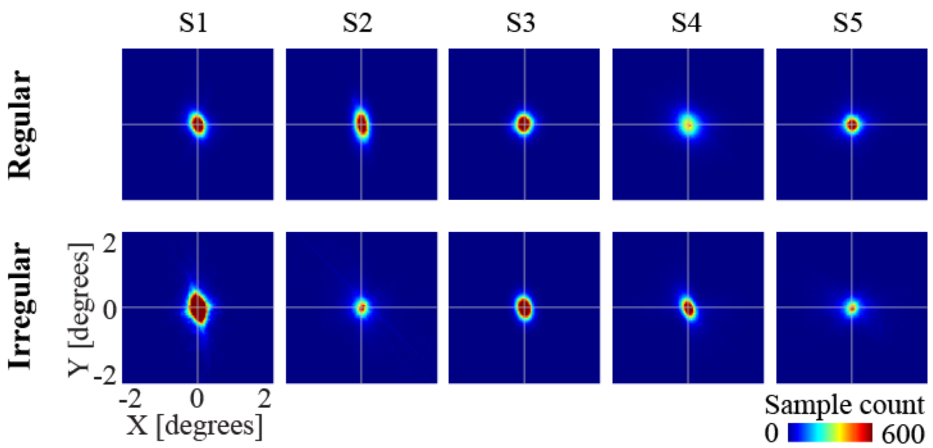

Figure S1. Eye movement results. Each subplot shows the averaged eye movement results of each subject from regular/irregular conditions.

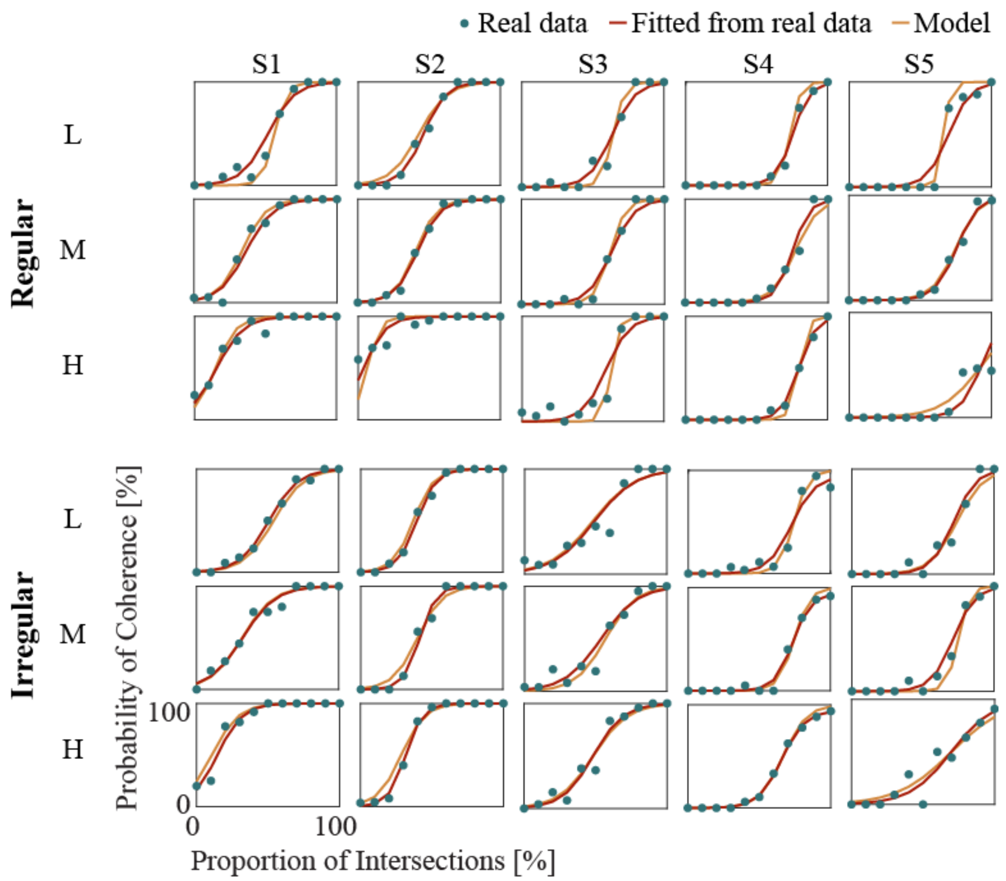

Figure S2. Psychometric functions. Empirical and simulated psychometric functions were plotted for each experiment and condition.

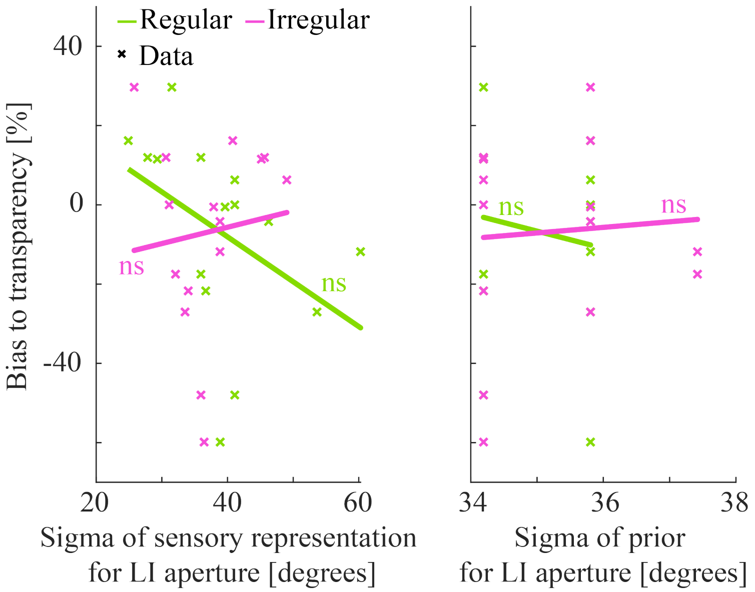

Figure S3. The amount of bias of transparent perception is not correlated with sensory representation of LI aperture (regular condition: r: -0.43, p: 0.10; irregular condition: r: 0.10, p: 0.70), nor with prior (regular condition: r: -0.14, p: 0.60; irregular condition: r: 0.06, p: 0.81)
